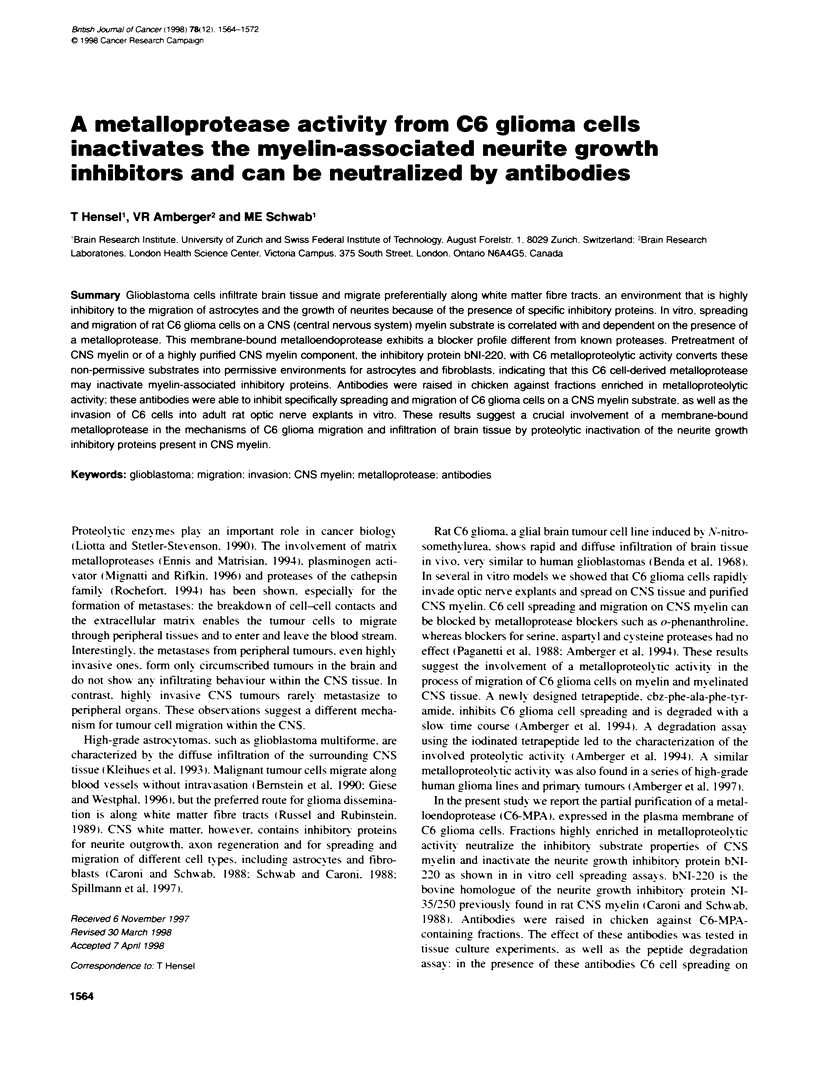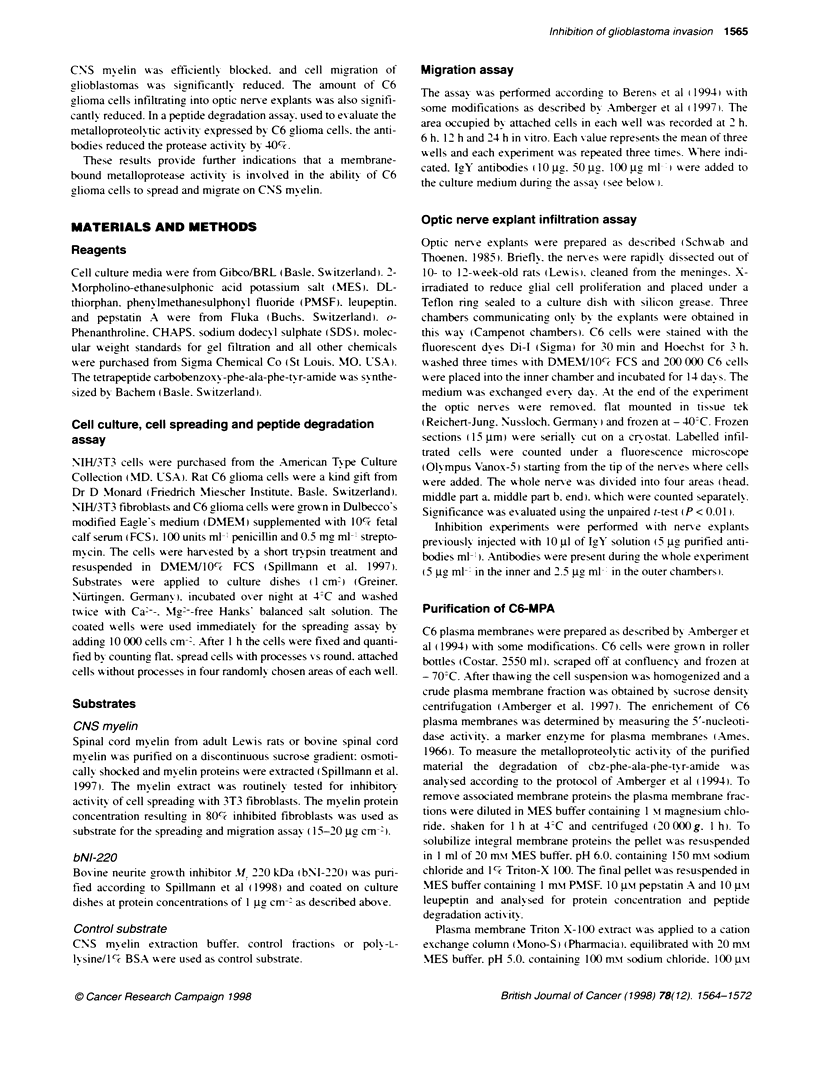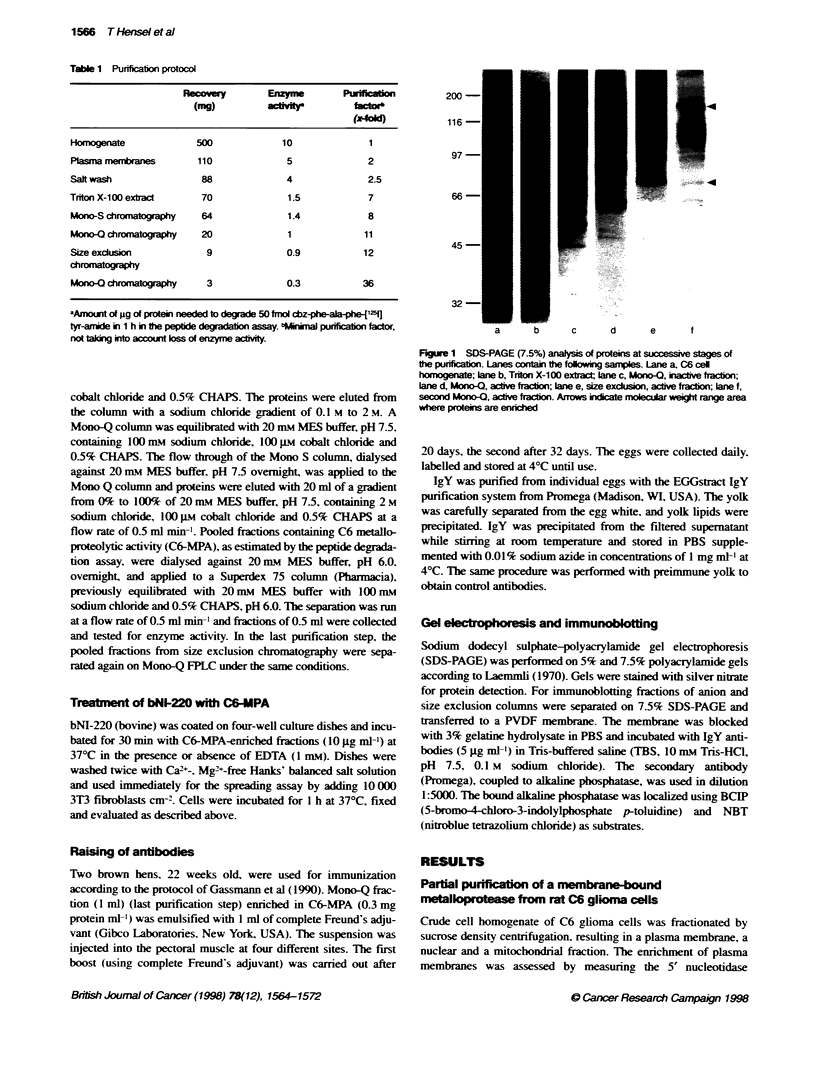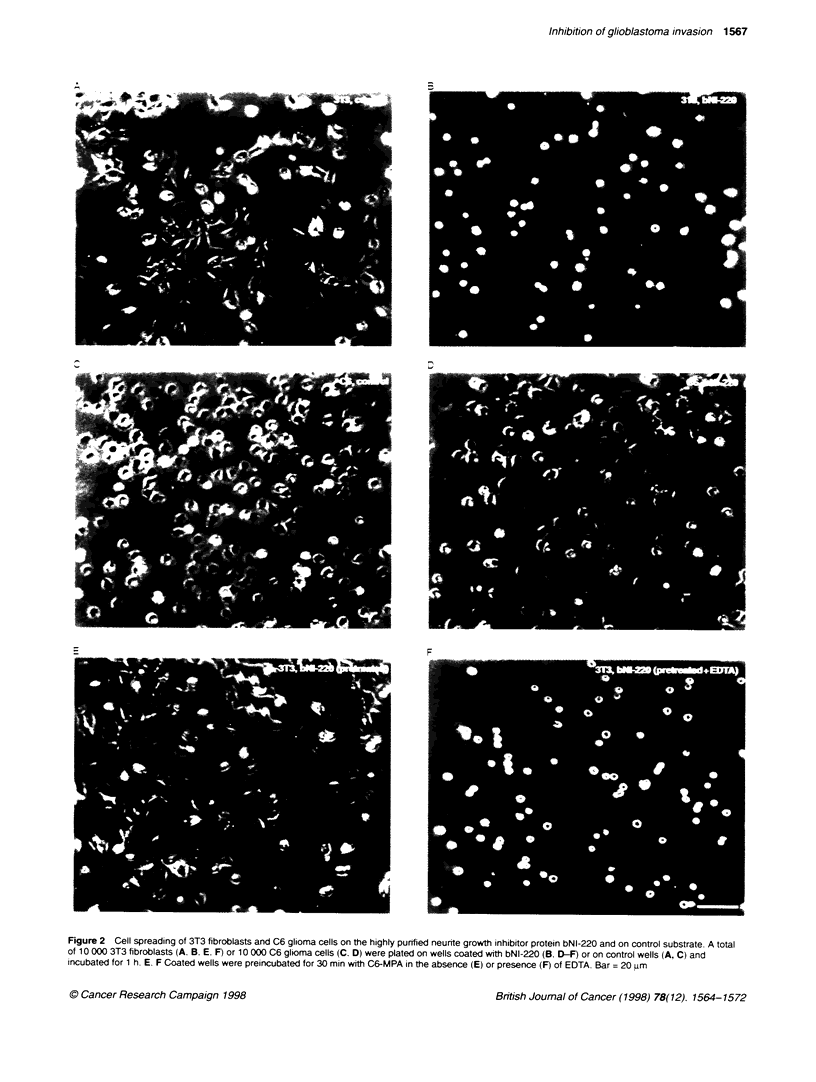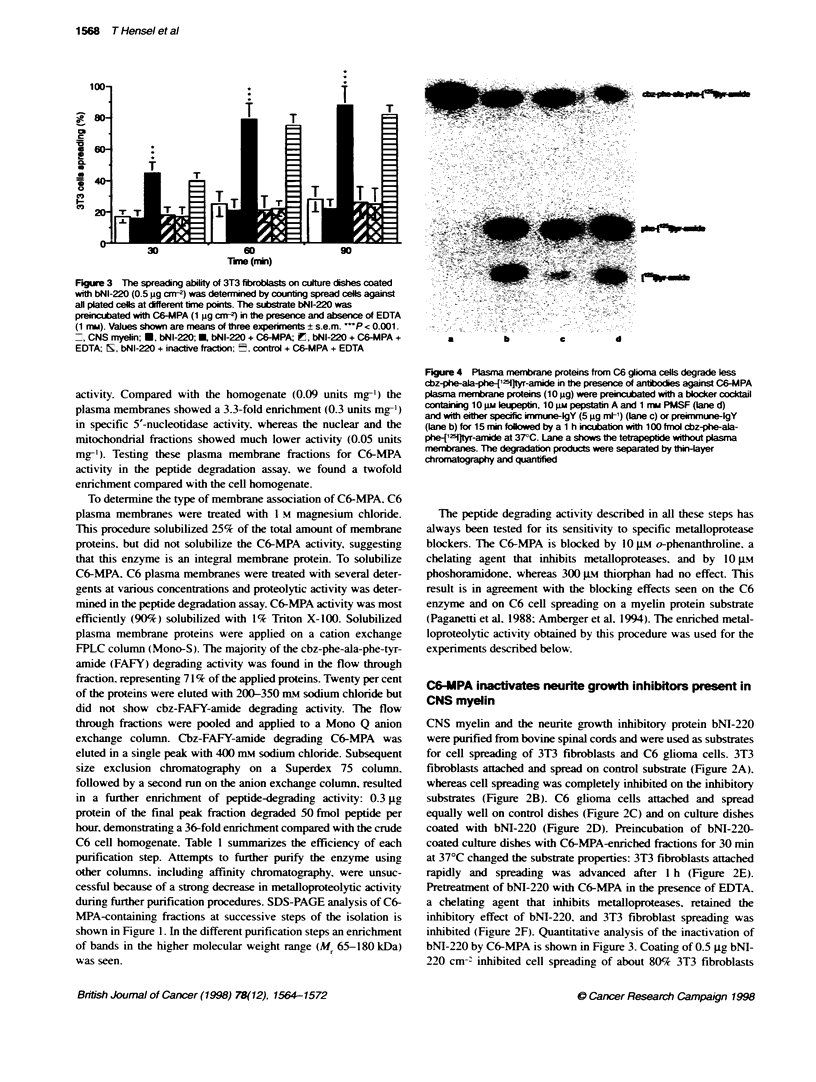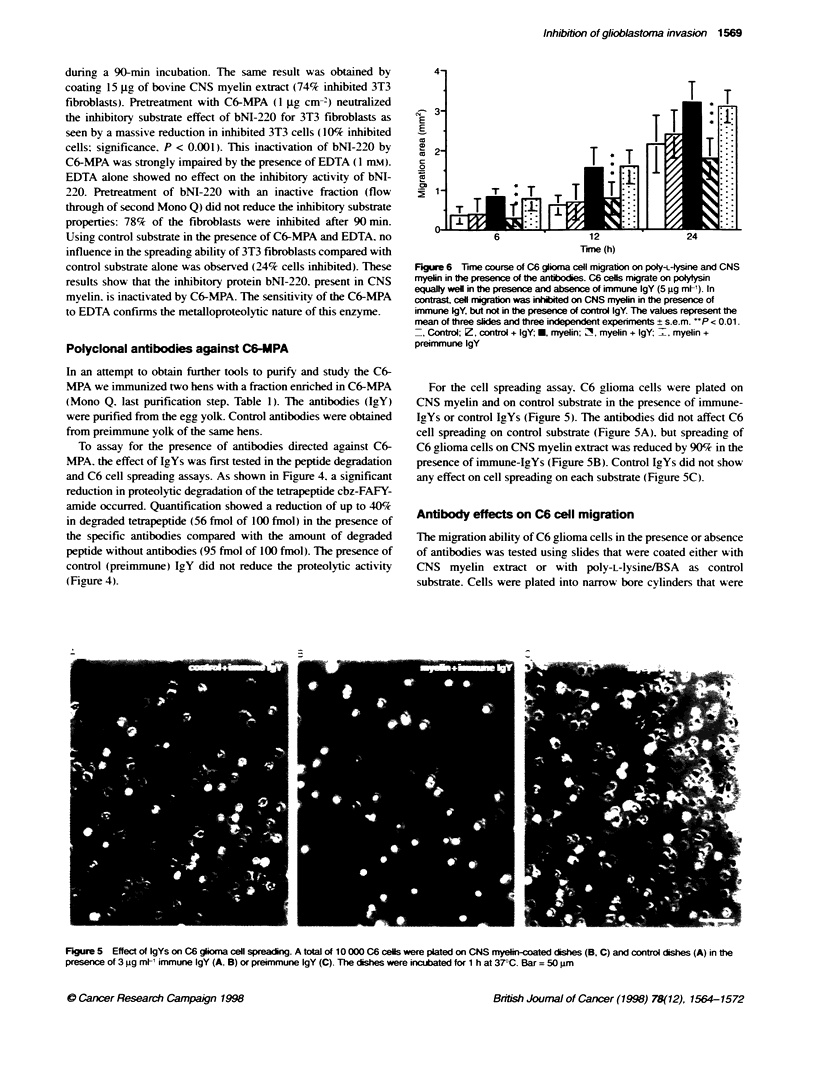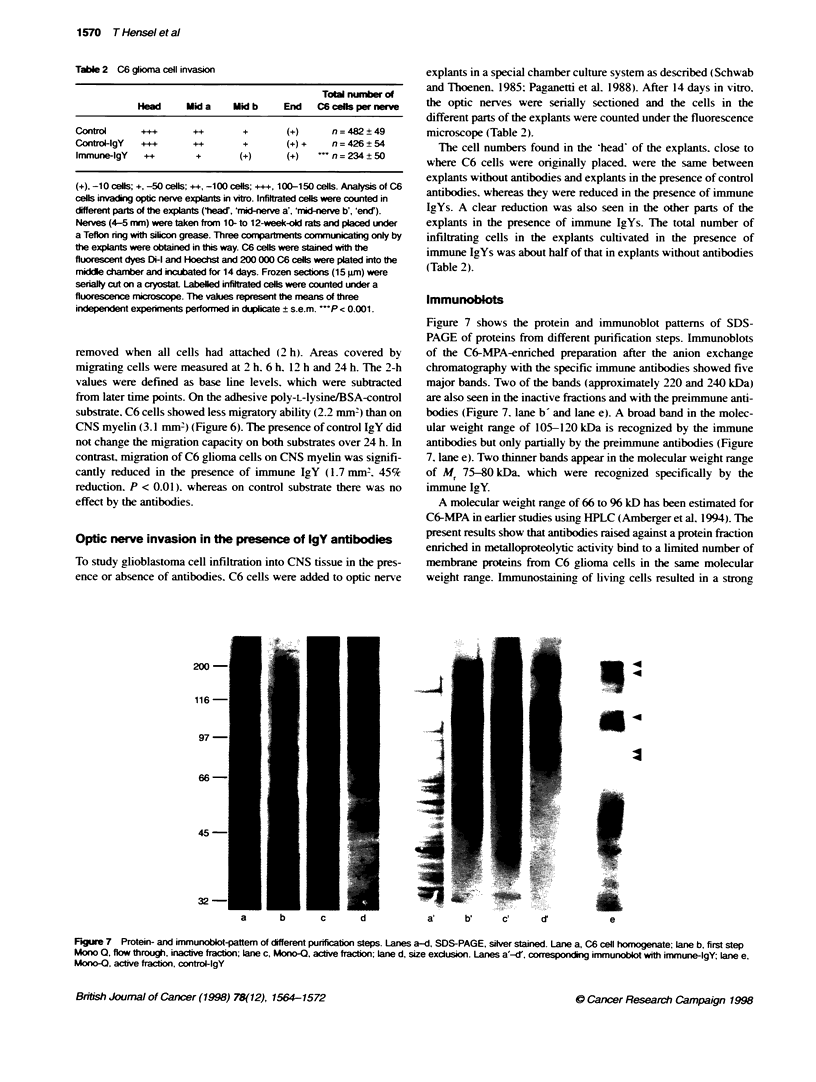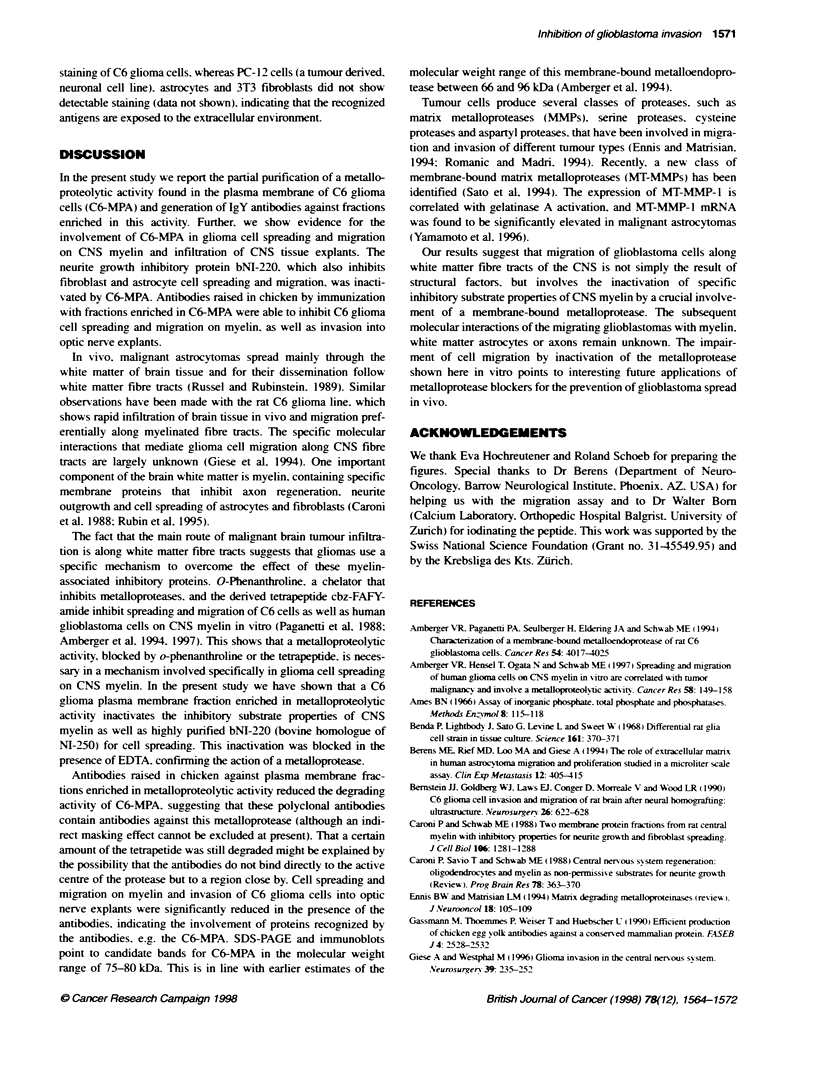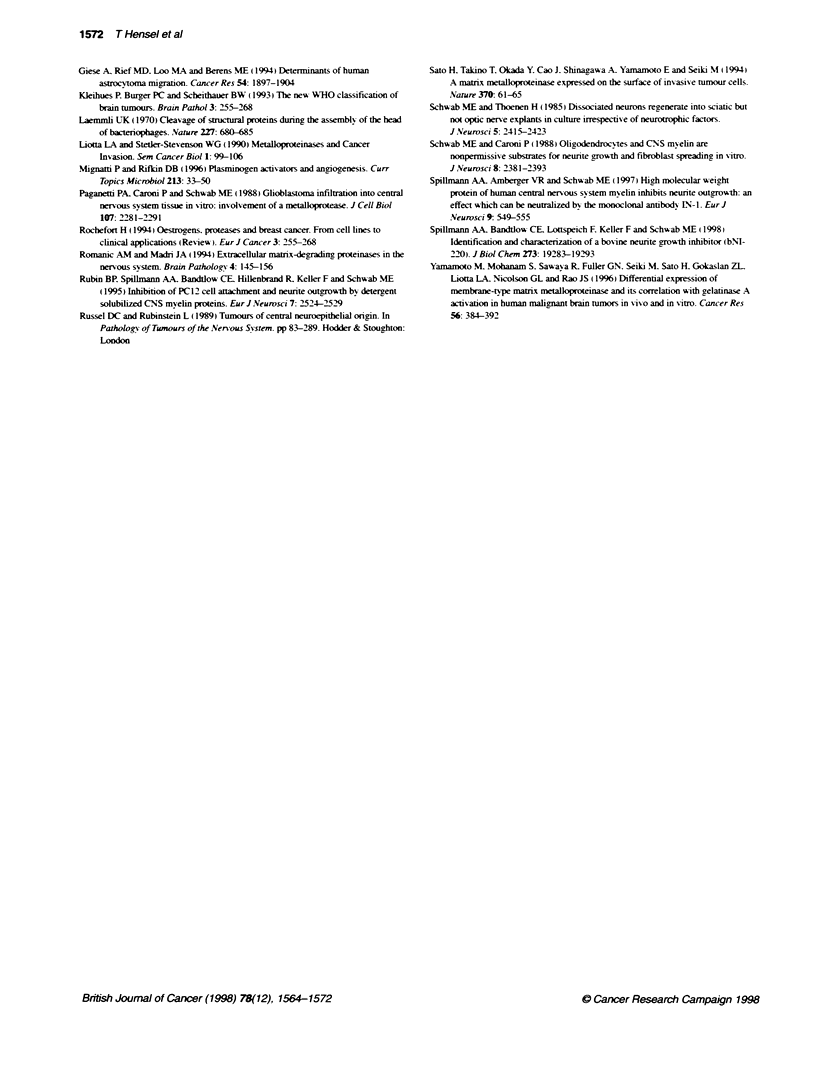# A metalloprotease activity from C6 glioma cells inactivates the myelin-associated neurite growth inhibitors and can be neutralized by antibodies.

**DOI:** 10.1038/bjc.1998.724

**Published:** 1998-12

**Authors:** T. Hensel, V. R. Amberger, M. E. Schwab

**Affiliations:** Brain Research Institute, University of Zurich and Swiss Federal Institute of Technology.

## Abstract

**Images:**


British Journal of Cancer (1998) 78(12), 1564–1572
© 1998 Cancer Research Campaign

### A metalloprotease activity from C6 glioma cells inactivates the myelin-associated neurite growth inhibitors and can be neutralized by antibodies

**T Hensel[1], VR Amberger[2] and ME Schwab[1]**

[1]Brain Research Institute, University of Zurich and Swiss Federal Institute of Technology, August Forelstr. 1, 8029 Zurich, Switzerland; [2]Brain Research Laboratories, London Health Science Center, Victoria Campus, 375 South Street, London, Ontario N6A4G5, Canada

**Summary** Glioblastoma cells infiltrate brain tissue and migrate preferentially along white matter fibre tracts, an environment that is highly inhibitory to the migration of astrocytes and the growth of neurites because of the presence of specific inhibitory proteins. In vitro, spreading and migration of rat C6 glioma cells on a CNS (central nervous system) myelin substrate is correlated with and dependent on the presence of a metalloprotease. This membrane-bound metalloendoprotease exhibits a blocker profile different from known proteases. Pretreatment of CNS myelin or of a highly purified CNS myelin component, the inhibitory protein bNI-220, with C6 metalloproteolytic activity converts these non-permissive substrates into permissive environments for astrocytes and fibroblasts, indicating that this C6 cell-derived metalloprotease may inactivate myelin-associated inhibitory proteins. Antibodies were raised in chicken against fractions enriched in metalloproteolytic activity; these antibodies were able to inhibit specifically spreading and migration of C6 glioma cells on a CNS myelin substrate, as well as the invasion of C6 cells into adult rat optic nerve explants in vitro. These results suggest a crucial involvement of a membrane-bound metalloprotease in the mechanisms of C6 glioma migration and infiltration of brain tissue by proteolytic inactivation of the neurite growth inhibitory proteins present in CNS myelin.

**Keywords:** glioblastoma; migration; invasion; CNS myelin; metalloprotease; antibodies

Proteolytic enzymes play an important role in cancer biology (Liotta and Stetler-Stevenson, 1990). The involvement of matrix metalloproteases (Ennis and Matrisian, 1994), plasminogen activator (Mignatti and Rifkin, 1996) and proteases of the cathepsin family (Rochefort, 1994) has been shown, especially for the formation of metastases: the breakdown of cell–cell contacts and the extracellular matrix enables the tumour cells to migrate through peripheral tissues and to enter and leave the blood stream. Interestingly, the metastases from peripheral tumours, even highly invasive ones, form only circumscribed tumours in the brain and do not show any infiltrating behaviour within the CNS tissue. In contrast, highly invasive CNS tumours rarely metastasize to peripheral organs. These observations suggest a different mechanism for tumour cell migration within the CNS.

High-grade astrocytomas, such as glioblastoma multiforme, are characterized by the diffuse infiltration of the surrounding CNS tissue (Kleihues et al, 1993). Malignant tumour cells migrate along blood vessels without intravasation (Bernstein et al, 1990; Giese and Westphal, 1996), but the preferred route for glioma dissemination is along white matter fibre tracts (Russel and Rubinstein, 1989). CNS white matter, however, contains inhibitory proteins for neurite outgrowth, axon regeneration and for spreading and migration of different cell types, including astrocytes and fibroblasts (Caroni and Schwab, 1988; Schwab and Caroni, 1988; Spillmann et al, 1997).

*Received 6 November 1997*
*Revised 30 March 1998*
*Accepted 7 April 1998*

*Correspondence to:* T Hensel

Rat C6 glioma, a glial brain tumour cell line induced by *N*-nitrosomethylurea, shows rapid and diffuse infiltration of brain tissue in vivo, very similar to human glioblastomas (Benda et al, 1968). In several in vitro models we showed that C6 glioma cells rapidly invade optic nerve explants and spread on CNS tissue and purified CNS myelin. C6 cell spreading and migration on CNS myelin can be blocked by metalloprotease blockers such as *o*-phenanthroline, whereas blockers for serine, aspartyl and cysteine proteases had no effect (Paganetti et al, 1988; Amberger et al, 1994). These results suggest the involvement of a metalloproteolytic activity in the process of migration of C6 glioma cells on myelin and myelinated CNS tissue. A newly designed tetrapeptide, cbz-phe-ala-phe-tyr-amide, inhibits C6 glioma cell spreading and is degraded with a slow time course (Amberger et al, 1994). A degradation assay using the iodinated tetrapeptide led to the characterization of the involved proteolytic activity (Amberger et al, 1994). A similar metalloproteolytic activity was also found in a series of high-grade human glioma lines and primary tumours (Amberger et al, 1997).

In the present study we report the partial purification of a metalloendoprotease (C6-MPA), expressed in the plasma membrane of C6 glioma cells. Fractions highly enriched in metalloproteolytic activity neutralize the inhibitory substrate properties of CNS myelin and inactivate the neurite growth inhibitory protein bNI-220 as shown in in vitro cell spreading assays. bNI-220 is the bovine homologue of the neurite growth inhibitory protein NI-35/250 previously found in rat CNS myelin (Caroni and Schwab, 1988). Antibodies were raised in chicken against C6-MPA-containing fractions. The effect of these antibodies was tested in tissue culture experiments, as well as the peptide degradation assay: in the presence of these antibodies C6 cell spreading on

CNS myelin was efficiently blocked, and cell migration of glioblastomas was significantly reduced. The amount of C6 glioma cells infiltrating into optic nerve explants was also significantly reduced. In a peptide degradation assay, used to evaluate the metalloproteolytic activity expressed by C6 glioma cells, the antibodies reduced the protease activity by 40%.

These results provide further indications that a membrane-bound metalloprotease activity is involved in the ability of C6 glioma cells to spread and migrate on CNS myelin.

### MATERIALS AND METHODS

### Reagents

Cell culture media were from Gibco/BRL (Basle, Switzerland). 2-Morpholino-ethanesulphonic acid potassium salt (MES), DL-thiorphan, phenylmethanesulphonyl fluoride (PMSF), leupeptin, and pepstatin A were from Fluka (Buchs, Switzerland). *o*-Phenanthroline, CHAPS, sodium dodecyl sulphate (SDS), molecular weight standards for gel filtration and all other chemicals were purchased from Sigma Chemical Co (St Louis, MO, USA). The tetrapeptide carbobenzoxy-phe-ala-phe-tyr-amide was synthesized by Bachem (Basle, Switzerland).

### Cell culture, cell spreading and peptide degradation assay

NIH/3T3 cells were purchased from the American Type Culture Collection (MD, USA). Rat C6 glioma cells were a kind gift from Dr D Monard (Friedrich Miescher Institute, Basle, Switzerland). NIH/3T3 fibroblasts and C6 glioma cells were grown in Dulbecco's modified Eagle's medium (DMEM) supplemented with 10% fetal calf serum (FCS), 100 units ml$^{-1}$ penicillin and 0.5 mg ml$^{-1}$ streptomycin. The cells were harvested by a short trypsin treatment and resuspended in DMEM/10% FCS (Spillmann et al, 1997). Substrates were applied to culture dishes (1 cm$^2$) (Greiner, Nürtingen, Germany), incubated over night at 4°C and washed twice with $Ca^{2+}$-, $Mg^{2+}$-free Hanks' balanced salt solution. The coated wells were used immediately for the spreading assay by adding 10 000 cells cm$^{-2}$. After 1 h the cells were fixed and quantified by counting flat, spread cells with processes vs round, attached cells without processes in four randomly chosen areas of each well.

### Substrates

#### *CNS myelin*

Spinal cord myelin from adult Lewis rats or bovine spinal cord myelin was purified on a discontinuous sucrose gradient; osmotically shocked and myelin proteins were extracted (Spillmann et al, 1997). The myelin extract was routinely tested for inhibitory activity of cell spreading with 3T3 fibroblasts. The myelin protein concentration resulting in 80% inhibited fibroblasts was used as substrate for the spreading and migration assay (15–20 µg cm$^{-2}$).

#### *bNI-220*

Bovine neurite growth inhibitor $M_r$ 220 kDa (bNI-220) was purified according to Spillmann et al (1998) and coated on culture dishes at protein concentrations of 1 µg cm$^{-2}$ as described above.

#### *Control substrate*

CNS myelin extraction buffer, control fractions or poly-L-lysine/1% BSA were used as control substrate.

### Migration assay

The assay was performed according to Berens et al (1994) with some modifications as described by Amberger et al (1997). The area occupied by attached cells in each well was recorded at 2 h, 6 h, 12 h and 24 h in vitro. Each value represents the mean of three wells and each experiment was repeated three times. Where indicated, IgY antibodies (10 µg, 50 µg, 100 µg ml$^{-1}$) were added to the culture medium during the assay (see below).

### Optic nerve explant infiltration assay

Optic nerve explants were prepared as described (Schwab and Thoenen, 1985). Briefly, the nerves were rapidly dissected out of 10- to 12-week-old rats (Lewis), cleaned from the meninges, X-irradiated to reduce glial cell proliferation and placed under a Teflon ring sealed to a culture dish with silicon grease. Three chambers communicating only by the explants were obtained in this way (Campenot chambers). C6 cells were stained with the fluorescent dyes Di-I (Sigma) for 30 min and Hoechst for 3 h, washed three times with DMEM/10% FCS and 200 000 C6 cells were placed into the inner chamber and incubated for 14 days. The medium was exchanged every day. At the end of the experiment the optic nerves were removed, flat mounted in tissue tek (Reichert-Jung, Nussloch, Germany) and frozen at – 40°C. Frozen sections (15 µm) were serially cut on a cryostat. Labelled infiltrated cells were counted under a fluorescence microscope (Olympus Vanox-5) starting from the tip of the nerves where cells were added. The whole nerve was divided into four areas (head, middle part a, middle part b, end), which were counted separately. Significance was evaluated using the unpaired *t*-test ($P < 0.01$).

Inhibition experiments were performed with nerve explants previously injected with 10 µl of IgY solution (5 µg purified antibodies ml$^{-1}$). Antibodies were present during the whole experiment (5 µg ml$^{-1}$ in the inner and 2.5 µg ml$^{-1}$ in the outer chambers).

### Purification of C6-MPA

C6 plasma membranes were prepared as described by Amberger et al (1994) with some modifications. C6 cells were grown in roller bottles (Costar, 2550 ml), scraped off at confluency and frozen at – 70°C. After thawing the cell suspension was homogenized and a crude plasma membrane fraction was obtained by sucrose density centrifugation (Amberger et al, 1997). The enrichement of C6 plasma membranes was determined by measuring the 5′-nucleotidase activity, a marker enzyme for plasma membranes (Ames, 1966). To measure the metalloproteolytic activity of the purified material the degradation of cbz-phe-ala-phe-tyr-amide was analysed according to the protocol of Amberger et al (1994). To remove associated membrane proteins the plasma membrane fractions were diluted in MES buffer containing 1 M magnesium chloride, shaken for 1 h at 4°C and centrifuged (20 000 *g*, 1 h). To solubilize integral membrane proteins the pellet was resuspended in 1 ml of 20 mM MES buffer, pH 6.0, containing 150 mM sodium chloride and 1% Triton-X 100. The final pellet was resuspended in MES buffer containing 1 mM PMSF, 10 µM pepstatin A and 10 µM leupeptin and analysed for protein concentration and peptide degradation activity.

Plasma membrane Triton X-100 extract was applied to a cation exchange column (Mono-S) (Pharmacia), equilibrated with 20 mM MES buffer, pH 5.0, containing 100 mM sodium chloride, 100 µM

© Cancer Research Campaign 1998

**Table 1** Purification protocol

| | Recovery (mg) | Enzyme activity[a] | Purification factor[b] (x-fold) |
|---|---|---|---|
| Homogenate | 500 | 10 | 1 |
| Plasma membranes | 110 | 5 | 2 |
| Salt wash | 88 | 4 | 2.5 |
| Triton X-100 extract | 70 | 1.5 | 7 |
| Mono-S chromatography | 64 | 1.4 | 8 |
| Mono-Q chromatography | 20 | 1 | 11 |
| Size exclusion chromatography | 9 | 0.9 | 12 |
| Mono-Q chromatography | 3 | 0.3 | 36 |

[a]Amount of μg of protein needed to degrade 50 fmol cbz-phe-ala-phe-[$^{125}$I] tyr-amide in 1 h in the peptide degradation assay. [b]Minimal purification factor, not taking into account loss of enzyme activity.

cobalt chloride and 0.5% CHAPS. The proteins were eluted from the column with a sodium chloride gradient of 0.1 M to 2 M. A Mono-Q column was equilibrated with 20 mM MES buffer, pH 7.5, containing 100 mM sodium chloride, 100 μM cobalt chloride and 0.5% CHAPS. The flow through of the Mono S column, dialysed against 20 mM MES buffer, pH 7.5 overnight, was applied to the Mono Q column and proteins were eluted with 20 ml of a gradient from 0% to 100% of 20 mM MES buffer, pH 7.5, containing 2 M sodium chloride, 100 μM cobalt chloride and 0.5% CHAPS at a flow rate of 0.5 ml min$^{-1}$. Pooled fractions containing C6 metalloproteolytic activity (C6-MPA), as estimated by the peptide degradation assay, were dialysed against 20 mM MES buffer, pH 6.0, overnight, and applied to a Superdex 75 column (Pharmacia), previously equilibrated with 20 mM MES buffer with 100 mM sodium chloride and 0.5% CHAPS, pH 6.0. The separation was run at a flow rate of 0.5 ml min$^{-1}$ and fractions of 0.5 ml were collected and tested for enzyme activity. In the last purification step, the pooled fractions from size exclusion chromatography were separated again on Mono-Q FPLC under the same conditions.

### Treatment of bNI-220 with C6-MPA

bNI-220 (bovine) was coated on four-well culture dishes and incubated for 30 min with C6-MPA-enriched fractions (10 μg ml$^{-1}$) at 37°C in the presence or absence of EDTA (1 mM). Dishes were washed twice with $Ca^{2+}$-, $Mg^{2+}$-free Hanks' balanced salt solution and used immediately for the spreading assay by adding 10 000 3T3 fibroblasts cm$^{-2}$. Cells were incubated for 1 h at 37°C, fixed and evaluated as described above.

### Raising of antibodies

Two brown hens, 22 weeks old, were used for immunization according to the protocol of Gassmann et al (1990). Mono-Q fraction (1 ml) (last purification step) enriched in C6-MPA (0.3 mg protein ml$^{-1}$) was emulsified with 1 ml of complete Freund's adjuvant (Gibco Laboratories, New York, USA). The suspension was injected into the pectoral muscle at four different sites. The first boost (using complete Freund's adjuvant) was carried out after

**Figure 1** SDS-PAGE (7.5%) analysis of proteins at successive stages of the purification. Lanes contain the following samples. Lane a, C6 cell homogenate; lane b, Triton X-100 extract; lane c, Mono-Q, inactive fraction; lane d, Mono-Q, active fraction; lane e, size exclusion, active fraction; lane f, second Mono-Q, active fraction. Arrows indicate molecular weight range area where proteins are enriched

20 days, the second after 32 days. The eggs were collected daily, labelled and stored at 4°C until use.

IgY was purified from individual eggs with the EGGstract IgY purification system from Promega (Madison, WI, USA). The yolk was carefully separated from the egg white, and yolk lipids were precipitated. IgY was precipitated from the filtered supernatant while stirring at room temperature and stored in PBS supplemented with 0.01% sodium azide in concentrations of 1 mg ml$^{-1}$ at 4°C. The same procedure was performed with preimmune yolk to obtain control antibodies.

### Gel electrophoresis and immunoblotting

Sodium dodecyl sulphate–polyacrylamide gel electrophoresis (SDS-PAGE) was performed on 5% and 7.5% polyacrylamide gels according to Laemmli (1970). Gels were stained with silver nitrate for protein detection. For immunoblotting fractions of anion and size exclusion columns were separated on 7.5% SDS-PAGE and transferred to a PVDF membrane. The membrane was blocked with 3% gelatine hydrolysate in PBS and incubated with IgY antibodies (5 μg ml$^{-1}$) in Tris-buffered saline (TBS, 10 mM Tris-HCl, pH 7.5, 0.1 M sodium chloride). The secondary antibody (Promega), coupled to alkaline phosphatase, was used in dilution 1:5000. The bound alkaline phosphatase was localized using BCIP (5-bromo-4-chloro-3-indolylphosphate *p*-toluidine) and NBT (nitroblue tetrazolium chloride) as substrates.

### RESULTS

### Partial purification of a membrane-bound metalloprotease from rat C6 glioma cells

Crude cell homogenate of C6 glioma cells was fractionated by sucrose density centrifugation, resulting in a plasma membrane, a nuclear and a mitochondrial fraction. The enrichment of plasma membranes was assessed by measuring the 5′ nucleotidase

© Cancer Research Campaign 1998

**Figure 2** Cell spreading of 3T3 fibroblasts and C6 glioma cells on the highly purified neurite growth inhibitor protein bNI-220 and on control substrate. A total of 10 000 3T3 fibroblasts (**A**. **B**. **E**. **F**) or 10 000 C6 glioma cells (**C**. **D**) were plated on wells coated with bNI-220 (**B**. **D**–**F**) or on control wells (**A**, **C**) and incubated for 1 h. **E**. **F** Coated wells were preincubated for 30 min with C6-MPA in the absence (**E**) or presence (**F**) of EDTA. Bar = 20 μm

*© Cancer Research Campaign 1998*

**Figure 3** The spreading ability of 3T3 fibroblasts on culture dishes coated with bNI-220 (0.5 µg cm$^{-2}$) was determined by counting spread cells against all plated cells at different time points. The substrate bNI-220 was preincubated with C6-MPA (1 µg cm$^{-2}$) in the presence and absence of EDTA (1 mM). Values shown are means of three experiments ± s.e.m. ***$P < 0.001$. □, CNS myelin; ■, bNI-220; ■, bNI-220 + C6-MPA; ▨, bNI-220 + C6-MPA + EDTA; ▧, bNI-220 + inactive fraction; ☰, control + C6-MPA + EDTA

**Figure 4** Plasma membrane proteins from C6 glioma cells degrade less cbz-phe-ala-phe-[$^{125}$I]tyr-amide in the presence of antibodies against C6-MPA plasma membrane proteins (10 µg) were preincubated with a blocker cocktail containing 10 µM leupeptin, 10 µM pepstatin A and 1 mM PMSF (lane d) and with either specific immune-IgY (5 µg ml$^{-1}$) (lane c) or preimmune-IgY (lane b) for 15 min followed by a 1 h incubation with 100 fmol cbz-phe-ala-phe-[$^{125}$I]tyr-amide at 37°C. Lane a shows the tetrapeptide without plasma membranes. The degradation products were separated by thin-layer chromatography and quantified activity. Compared with the homogenate (0.09 units mg$^{-1}$) the plasma membranes showed a 3.3-fold enrichment (0.3 units mg$^{-1}$) in specific 5′-nucleotidase activity, whereas the nuclear and the mitochondrial fractions showed much lower activity (0.05 units mg$^{-1}$). Testing these plasma membrane fractions for C6-MPA activity in the peptide degradation assay, we found a twofold enrichment compared with the cell homogenate.

To determine the type of membrane association of C6-MPA, C6 plasma membranes were treated with 1 M magnesium chloride. This procedure solubilized 25% of the total amount of membrane proteins, but did not solubilize the C6-MPA activity, suggesting that this enzyme is an integral membrane protein. To solubilize C6-MPA, C6 plasma membranes were treated with several detergents at various concentrations and proteolytic activity was determined in the peptide degradation assay. C6-MPA activity was most efficiently (90%) solubilized with 1% Triton X-100. Solubilized plasma membrane proteins were applied on a cation exchange FPLC column (Mono-S). The majority of the cbz-phe-ala-phe-tyr-amide (FAFY) degrading activity was found in the flow through fraction, representing 71% of the applied proteins. Twenty per cent of the proteins were eluted with 200–350 mM sodium chloride but did not show cbz-FAFY-amide degrading activity. The flow through fractions were pooled and applied to a Mono Q anion exchange column. Cbz-FAFY-amide degrading C6-MPA was eluted in a single peak with 400 mM sodium chloride. Subsequent size exclusion chromatography on a Superdex 75 column, followed by a second run on the anion exchange column, resulted in a further enrichment of peptide-degrading activity: 0.3 µg protein of the final peak fraction degraded 50 fmol peptide per hour, demonstrating a 36-fold enrichment compared with the crude C6 cell homogenate. Table 1 summarizes the efficiency of each purification step. Attempts to further purify the enzyme using other columns, including affinity chromatography, were unsuccessful because of a strong decrease in metalloproteolytic activity during further purification procedures. SDS-PAGE analysis of C6-MPA-containing fractions at successive steps of the isolation is shown in Figure 1. In the different purification steps an enrichment of bands in the higher molecular weight range ($M_r$ 65–180 kDa) was seen.

The peptide degrading activity described in all these steps has always been tested for its sensitivity to specific metalloprotease blockers. The C6-MPA is blocked by 10 µM *o*-phenanthroline, a chelating agent that inhibits metalloproteases, and by 10 µM phoshoramidone, whereas 300 µM thiorphan had no effect. This result is in agreement with the blocking effects seen on the C6 enzyme and on C6 cell spreading on a myelin protein substrate (Paganetti et al, 1988; Amberger et al, 1994). The enriched metalloproteolytic activity obtained by this procedure was used for the experiments described below.

### C6-MPA inactivates neurite growth inhibitors present in CNS myelin

CNS myelin and the neurite growth inhibitory protein bNI-220 were purified from bovine spinal cords and were used as substrates for cell spreading of 3T3 fibroblasts and C6 glioma cells. 3T3 fibroblasts attached and spread on control substrate (Figure 2A), whereas cell spreading was completely inhibited on the inhibitory substrates (Figure 2B). C6 glioma cells attached and spread equally well on control dishes (Figure 2C) and on culture dishes coated with bNI-220 (Figure 2D). Preincubation of bNI-220-coated culture dishes with C6-MPA-enriched fractions for 30 min at 37°C changed the substrate properties: 3T3 fibroblasts attached rapidly and spreading was advanced after 1 h (Figure 2E). Pretreatment of bNI-220 with C6-MPA in the presence of EDTA, a chelating agent that inhibits metalloproteases, retained the inhibitory effect of bNI-220, and 3T3 fibroblast spreading was inhibited (Figure 2F). Quantitative analysis of the inactivation of bNI-220 by C6-MPA is shown in Figure 3. Coating of 0.5 µg bNI-220 cm$^{-2}$ inhibited cell spreading of about 80% 3T3 fibroblasts

 © Cancer Research Campaign 1998

during a 90-min incubation. The same result was obtained by coating 15 µg of bovine CNS myelin extract (74% inhibited 3T3 fibroblasts). Pretreatment with C6-MPA (1 µg $cm^{-2}$) neutralized the inhibitory substrate effect of bNI-220 for 3T3 fibroblasts as seen by a massive reduction in inhibited 3T3 cells (10% inhibited cells; significance, $P < 0.001$). This inactivation of bNI-220 by C6-MPA was strongly impaired by the presence of EDTA (1 mM). EDTA alone showed no effect on the inhibitory activity of bNI-220. Pretreatment of bNI-220 with an inactive fraction (flow through of second Mono Q) did not reduce the inhibitory substrate properties: 78% of the fibroblasts were inhibited after 90 min. Using control substrate in the presence of C6-MPA and EDTA, no influence in the spreading ability of 3T3 fibroblasts compared with control substrate alone was observed (24% cells inhibited). These results show that the inhibitory protein bNI-220, present in CNS myelin, is inactivated by C6-MPA. The sensitivity of the C6-MPA to EDTA confirms the metalloproteolytic nature of this enzyme.

### Polyclonal antibodies against C6-MPA

In an attempt to obtain further tools to purify and study the C6-MPA we immunized two hens with a fraction enriched in C6-MPA (Mono Q, last purification step, Table 1). The antibodies (IgY) were purified from the egg yolk. Control antibodies were obtained from preimmune yolk of the same hens.

To assay for the presence of antibodies directed against C6-MPA, the effect of IgYs was first tested in the peptide degradation and C6 cell spreading assays. As shown in Figure 4, a significant reduction in proteolytic degradation of the tetrapeptide cbz-FAFY-amide occurred. Quantification showed a reduction of up to 40% in degraded tetrapeptide (56 fmol of 100 fmol) in the presence of the specific antibodies compared with the amount of degraded peptide without antibodies (95 fmol of 100 fmol). The presence of control (preimmune) IgY did not reduce the proteolytic activity (Figure 4).

**Figure 6** Time course of C6 glioma cell migration on poly-L-lysine and CNS myelin in the presence of the antibodies. C6 cells migrate on polylysin equally well in the presence and absence of immune IgY (5 µg $ml^{-1}$). In contrast, cell migration was inhibited on CNS myelin in the presence of immune IgY, but not in the presence of control IgY. The values represent the mean of three slides and three independent experiments ± s.e.m. $^{**}P < 0.01$. ☐, Control; ▨, control + IgY; ■, myelin; ▧, myelin + IgY; ▤, myelin + preimmune IgY

For the cell spreading assay, C6 glioma cells were plated on CNS myelin and on control substrate in the presence of immune-IgYs or control IgYs (Figure 5). The antibodies did not affect C6 cell spreading on control substrate (Figure 5A), but spreading of C6 glioma cells on CNS myelin extract was reduced by 90% in the presence of immune-IgYs (Figure 5B). Control IgYs did not show any effect on cell spreading on each substrate (Figure 5C).

### Antibody effects on C6 cell migration

The migration ability of C6 glioma cells in the presence or absence of antibodies was tested using slides that were coated either with CNS myelin extract or with poly-L-lysine/BSA as control substrate. Cells were plated into narrow bore cylinders that were

**Figure 5** Effect of IgYs on C6 glioma cell spreading. A total of 10 000 C6 cells were plated on CNS myelin-coated dishes (**B**, **C**) and control dishes (**A**) in the presence of 3 µg $ml^{-1}$ immune IgY (**A**, **B**) or preimmune IgY (**C**). The dishes were incubated for 1 h at 37°C. Bar = 50 µm

© Cancer Research Campaign 1998

**Table 2** C6 glioma cell invasion

| | Head | Mid a | Mid b | End | Total number of C6 cells per nerve |
|---|---|---|---|---|---|
| Control | +++ | ++ | + | (+) | $n = 482 \pm 49$ |
| Control-IgY | +++ | ++ | + | (+) + | $n = 426 \pm 54$ |
| Immune-IgY | ++ | + | (+) | (+) | *** $n = 234 \pm 50$ |

(+), −10 cells; +, −50 cells; ++, −100 cells; +++, 100–150 cells. Analysis of C6 cells invading optic nerve explants in vitro. Infiltrated cells were counted in different parts of the explants ('head', 'mid-nerve a', 'mid-nerve b', 'end'). Nerves (4–5 mm) were taken from 10- to 12-week-old rats and placed under a Teflon ring with silicon grease. Three compartments communicating only by the explants were obtained in this way. C6 cells were stained with the fluorescent dyes Di-I and Hoechst and 200 000 C6 cells were plated into the middle chamber and incubated for 14 days. Frozen sections (15 μm) were serially cut on a cryostat. Labelled infiltrated cells were counted under a fluorescence microscope. The values represent the means of three independent experiments performed in duplicate ± s.e.m. ***$P < 0.001$.

removed when all cells had attached (2 h). Areas covered by migrating cells were measured at 2 h, 6 h, 12 h and 24 h. The 2-h values were defined as base line levels, which were subtracted from later time points. On the adhesive poly-L-lysine/BSA-control substrate, C6 cells showed less migratory ability (2.2 $mm^2$) than on CNS myelin (3.1 $mm^2$) (Figure 6). The presence of control IgY did not change the migration capacity on both substrates over 24 h. In contrast, migration of C6 glioma cells on CNS myelin was significantly reduced in the presence of immune IgY (1.7 $mm^2$, 45% reduction, $P < 0.01$), whereas on control substrate there was no effect by the antibodies.

### Optic nerve invasion in the presence of IgY antibodies

To study glioblastoma cell infiltration into CNS tissue in the presence or absence of antibodies, C6 cells were added to optic nerve explants in a special chamber culture system as described (Schwab and Thoenen, 1985; Paganetti et al, 1988). After 14 days in vitro, the optic nerves were serially sectioned and the cells in the different parts of the explants were counted under the fluorescence microscope (Table 2).

The cell numbers found in the 'head' of the explants, close to where C6 cells were originally placed, were the same between explants without antibodies and explants in the presence of control antibodies, whereas they were reduced in the presence of immune IgYs. A clear reduction was also seen in the other parts of the explants in the presence of immune IgYs. The total number of infiltrating cells in the explants cultivated in the presence of immune IgYs was about half of that in explants without antibodies (Table 2).

### Immunoblots

Figure 7 shows the protein and immunoblot patterns of SDS-PAGE of proteins from different purification steps. Immunoblots of the C6-MPA-enriched preparation after the anion exchange chromatography with the specific immune antibodies showed five major bands. Two of the bands (approximately 220 and 240 kDa) are also seen in the inactive fractions and with the preimmune antibodies (Figure 7, lane b′ and lane e). A broad band in the molecular weight range of 105–120 kDa is recognized by the immune antibodies but only partially by the preimmune antibodies (Figure 7, lane e). Two thinner bands appear in the molecular weight range of $M_r$ 75–80 kDa, which were recognized specifically by the immune IgY.

A molecular weight range of 66 to 96 kD has been estimated for C6-MPA in earlier studies using HPLC (Amberger et al, 1994). The present results show that antibodies raised against a protein fraction enriched in metalloproteolytic activity bind to a limited number of membrane proteins from C6 glioma cells in the same molecular weight range. Immunostaining of living cells resulted in a strong

**Figure 7** Protein- and immunoblot-pattern of different purification steps. Lanes a–d, SDS-PAGE, silver stained. Lane a, C6 cell homogenate; lane b, first step Mono Q, flow through, inactive fraction; lane c, Mono-Q, active fraction; lane d, size exclusion. Lanes a′–d′, corresponding immunoblot with immune-IgY; lane e, Mono-Q, active fraction, control-IgY

 © Cancer Research Campaign 1998

staining of C6 glioma cells, whereas PC-12 cells (a tumour derived, neuronal cell line), astrocytes and 3T3 fibroblasts did not show detectable staining (data not shown), indicating that the recognized antigens are exposed to the extracellular environment.

### DISCUSSION

In the present study we report the partial purification of a metalloproteolytic activity found in the plasma membrane of C6 glioma cells (C6-MPA) and generation of IgY antibodies against fractions enriched in this activity. Further, we show evidence for the involvement of C6-MPA in glioma cell spreading and migration on CNS myelin and infiltration of CNS tissue explants. The neurite growth inhibitory protein bNI-220, which also inhibits fibroblast and astrocyte cell spreading and migration, was inactivated by C6-MPA. Antibodies raised in chicken by immunization with fractions enriched in C6-MPA were able to inhibit C6 glioma cell spreading and migration on myelin, as well as invasion into optic nerve explants.

In vivo, malignant astrocytomas spread mainly through the white matter of brain tissue and for their dissemination follow white matter fibre tracts (Russel and Rubinstein, 1989). Similar observations have been made with the rat C6 glioma line, which shows rapid infiltration of brain tissue in vivo and migration preferentially along myelinated fibre tracts. The specific molecular interactions that mediate glioma cell migration along CNS fibre tracts are largely unknown (Giese et al, 1994). One important component of the brain white matter is myelin, containing specific membrane proteins that inhibit axon regeneration, neurite outgrowth and cell spreading of astrocytes and fibroblasts (Caroni et al, 1988; Rubin et al, 1995).

The fact that the main route of malignant brain tumour infiltration is along white matter fibre tracts suggests that gliomas use a specific mechanism to overcome the effect of these myelin-associated inhibitory proteins. *O*-Phenanthroline, a chelator that inhibits metalloproteases, and the derived tetrapeptide cbz-FAFY-amide inhibit spreading and migration of C6 cells as well as human glioblastoma cells on CNS myelin in vitro (Paganetti et al, 1988; Amberger et al, 1994, 1997). This shows that a metalloproteolytic activity, blocked by *o*-phenanthroline or the tetrapeptide, is necessary in a mechanism involved specifically in glioma cell spreading on CNS myelin. In the present study we have shown that a C6 glioma plasma membrane fraction enriched in metalloproteolytic activity inactivates the inhibitory substrate properties of CNS myelin as well as highly purified bNI-220 (bovine homologue of NI-250) for cell spreading. This inactivation was blocked in the presence of EDTA, confirming the action of a metalloprotease.

Antibodies raised in chicken against plasma membrane fractions enriched in metalloproteolytic activity reduced the degrading activity of C6-MPA, suggesting that these polyclonal antibodies contain antibodies against this metalloprotease (although an indirect masking effect cannot be excluded at present). That a certain amount of the tetrapetide was still degraded might be explained by the possibility that the antibodies do not bind directly to the active centre of the protease but to a region close by. Cell spreading and migration on myelin and invasion of C6 glioma cells into optic nerve explants were significantly reduced in the presence of the antibodies, indicating the involvement of proteins recognized by the antibodies, e.g. the C6-MPA. SDS-PAGE and immunoblots point to candidate bands for C6-MPA in the molecular weight range of 75–80 kDa. This is in line with earlier estimates of the molecular weight range of this membrane-bound metalloendoprotease between 66 and 96 kDa (Amberger et al, 1994).

Tumour cells produce several classes of proteases, such as matrix metalloproteases (MMPs), serine proteases, cysteine proteases and aspartyl proteases, that have been involved in migration and invasion of different tumour types (Ennis and Matrisian, 1994; Romanic and Madri, 1994). Recently, a new class of membrane-bound matrix metalloproteases (MT-MMPs) has been identified (Sato et al, 1994). The expression of MT-MMP-1 is correlated with gelatinase A activation, and MT-MMP-1 mRNA was found to be significantly elevated in malignant astrocytomas (Yamamoto et al, 1996).

Our results suggest that migration of glioblastoma cells along white matter fibre tracts of the CNS is not simply the result of structural factors, but involves the inactivation of specific inhibitory substrate properties of CNS myelin by a crucial involvement of a membrane-bound metalloprotease. The subsequent molecular interactions of the migrating glioblastomas with myelin, white matter astrocytes or axons remain unknown. The impairment of cell migration by inactivation of the metalloprotease shown here in vitro points to interesting future applications of metalloprotease blockers for the prevention of glioblastoma spread in vivo.

### ACKNOWLEDGEMENTS

We thank Eva Hochreutener and Roland Schoeb for preparing the figures. Special thanks to Dr Berens (Department of Neuro-Oncology, Barrow Neurological Institute, Phoenix, AZ, USA) for helping us with the migration assay and to Dr Walter Born (Calcium Laboratory, Orthopedic Hospital Balgrist, University of Zurich) for iodinating the peptide. This work was supported by the Swiss National Science Foundation (Grant no. 31-45549.95) and by the Krebsliga des Kts. Zürich.

### REFERENCES

Amberger VR, Paganetti PA, Seulberger H, Eldering JA and Schwab ME (1994) Characterization of a membrane-bound metalloendoprotease of rat C6 glioblastoma cells. *Cancer Res* **54**: 4017–4025

Amberger VR, Hensel T, Ogata N and Schwab ME (1997) Spreading and migration of human glioma cells on CNS myelin in vitro are correlated with tumor malignancy and involve a metalloproteolytic activity. *Cancer Res* **58**: 149–158

Ames BN (1966) Assay of inorganic phosphate, total phosphate and phosphatases. *Methods Enzymol* **8**: 115–118

Benda P, Lightbody J, Sato G, Levine L and Sweet W (1968) Differential rat glia cell strain in tissue culture. *Science* **161**: 370–371

Berens ME, Rief MD, Loo MA and Giese A (1994) The role of extracellular matrix in human astrocytoma migration and proliferation studied in a microliter scale assay. *Clin Exp Metastasis* **12**: 405–415

Bernstein JJ, Goldberg WJ, Laws EJ, Conger D, Morreale V and Wood LR (1990) C6 glioma cell invasion and migration of rat brain after neural homografting: ultrastructure. *Neurosurgery* **26**: 622–628

Caroni P and Schwab ME (1988) Two membrane protein fractions from rat central myelin with inhibitory properties for neurite growth and fibroblast spreading. *J Cell Biol* **106**: 1281–1288

Caroni P, Savio T and Schwab ME (1988) Central nervous system regeneration: oligodendrocytes and myelin as non-permissive substrates for neurite growth (Review). *Prog Brain Res* **78**: 363–370

Ennis BW and Matrisian LM (1994) Matrix degrading metalloproteinases (review). *J Neurooncol* **18**: 105–109

Gassmann M, Thoemmes P, Weiser T and Huebscher U (1990) Efficient production of chicken egg yolk antibodies against a conserved mammalian protein. *FASEB J* **4**: 2528–2532

Giese A and Westphal M (1996) Glioma invasion in the central nervous system. *Neurosurgery* **39**: 235–252

© Cancer Research Campaign 1998 

Giese A. Rief MD. Loo MA and Berens ME (1994) Determinants of human astrocytoma migration. *Cancer Res* **54**: 1897–1904
Kleihues P. Burger PC and Scheithauer BW (1993) The new WHO classification of brain tumours. *Brain Pathol* **3**: 255–268
Laemmli UK (1970) Cleavage of structural proteins during the assembly of the head of bacteriophages. *Nature* **227**: 680–685
Liotta LA and Stetler-Stevenson WG (1990) Metalloproteinases and Cancer Invasion. *Sem Cancer Biol* **1**: 99–106
Mignatti P and Rifkin DB (1996) Plasminogen activators and angiogenesis. *Curr Topics Microbiol* **213**: 33–50
Paganetti PA. Caroni P and Schwab ME (1988) Glioblastoma infiltration into central nervous system tissue in vitro: involvement of a metalloprotease. *J Cell Biol* **107**: 2281–2291
Rochefort H (1994) Oestrogens, proteases and breast cancer. From cell lines to clinical applications (Review). *Eur J Cancer* **3**: 255–268
Romanic AM and Madri JA (1994) Extracellular matrix-degrading proteinases in the nervous system. *Brain Pathology* **4**: 145–156
Rubin BP. Spillmann AA. Bandtlow CE. Hillenbrand R. Keller F and Schwab ME (1995) Inhibition of PC12 cell attachment and neurite outgrowth by detergent solubilized CNS myelin proteins. *Eur J Neurosci* **7**: 2524–2529
Russel DC and Rubinstein L (1989) Tumours of central neuroepithelial origin. In *Pathology of Tumours of the Nervous System*. pp 83–289. Hodder & Stoughton: London
Sato H. Takino T. Okada Y. Cao J. Shinagawa A. Yamamoto E and Seiki M (1994) A matrix metalloproteinase expressed on the surface of invasive tumour cells. *Nature* **370**: 61–65
Schwab ME and Thoenen H (1985) Dissociated neurons regenerate into sciatic but not optic nerve explants in culture irrespective of neurotrophic factors. *J Neurosci* **5**: 2415–2423
Schwab ME and Caroni P (1988) Oligodendrocytes and CNS myelin are nonpermissive substrates for neurite growth and fibroblast spreading in vitro. *J Neurosci* **8**: 2381–2393
Spillmann AA. Amberger VR and Schwab ME (1997) High molecular weight protein of human central nervous system myelin inhibits neurite outgrowth: an effect which can be neutralized by the monoclonal antibody IN-1. *Eur J Neurosci* **9**: 549–555
Spillmann AA. Bandtlow CE. Lottspeich F. Keller F and Schwab ME (1998) Identification and characterization of a bovine neurite growth inhibitor (bNI-220). *J Biol Chem* **273**: 19283–19293
Yamamoto M. Mohanam S. Sawaya R. Fuller GN. Seiki M. Sato H. Gokaslan ZL. Liotta LA. Nicolson GL and Rao JS (1996) Differential expression of membrane-type matrix metalloproteinase and its correlation with gelatinase A activation in human malignant brain tumors in vivo and in vitro. *Cancer Res* **56**: 384–392